# Age and Gender Differences in Cardiovascular Autonomic Failure in the Transgenic PLP-syn Mouse, a Model of Multiple System Atrophy

**DOI:** 10.3389/fneur.2022.874155

**Published:** 2022-06-02

**Authors:** Marc Kermorgant, Pierre-Olivier Fernagut, Wassilios G. Meissner, Dina N. Arvanitis, Du N'Guyen, Jean-Michel Senard, Anne Pavy-Le Traon

**Affiliations:** ^1^INSERM DR Midi-Pyrénées Limousin, Institute of Cardiovascular and Metabolic Diseases (I2MC) UMR1297, University Hospital of Toulouse, Toulouse, France; ^2^French Reference Center for Multiple System Atrophy, Neurology Department, University Hospital of Toulouse, Toulouse, France; ^3^Univ. Bordeaux, CNRS, IMN, UMR 5293, Bordeaux, France; ^4^Laboratoire de Neurosciences Expérimentales et Cliniques INSERM U1084, University of Poitiers, Poitiers, France; ^5^CRMR AMS, Service de Neurologie - Maladies Neurodégénératives, CHU de Bordeaux, Bordeaux, France; ^6^Department of Medicine, University of Otago, Christchurch, New Zealand; ^7^New Zealand Brain Research Institute, Christchurch, New Zealand; ^8^Department of Clinical Pharmacology, University Hospital of Toulouse, Toulouse, France

**Keywords:** multiple system atrophy, alpha-synuclein, heart rate variability, baroreflex, QTc interval, cardiovascular autonomic failure, age, gender

## Abstract

Multiple system atrophy (MSA) is a rare and progressive neurodegenerative disorder. Autonomic failure (AF) is one main clinical feature which has a significant impact on health-related quality of life. The neuropathological hallmark of MSA is the abnormal accumulation of α-synuclein in oligodendrocytes forming glial cytoplasmic inclusions. Only little is known about gender and age differences in AF in MSA. This study was carried out in 6 and 12 months old transgenic PLP-α-syn and WT male and female mice. Heart rate variability (HRV) was assessed both in time, frequential and non-linear domains. Baroreflex sensitivity (BRS) was estimated by the sequence method. Duration of ventricular depolarization and repolarization (QT/QTc intervals) were evaluated from the ECG signals. Three-way ANOVA (genotype x gender x age) with Sidak's method *post-hoc* was used to analyze data. BRS was significantly changed in PLP-α-syn mice and was age-dependent. QT and QTc intervals were not significantly modified in PLP-α-syn mice. An impaired HRV was observed at 12 months of age in PLP-α-syn female but not in male mice, indicative of cardiovascular AF.

## Introduction

Multiple system atrophy (MSA) is a progressive, sporadic and adult-onset neurodegenerative disease characterized by a combination of autonomic failure with parkinsonism, cerebellar ataxia and autonomic dysfunction (1). Cardiovascular autonomic failure (AF) is one of the main features which worsens dramatically the quality of life. MSA is characterized by an abnormal accumulation of α-synuclein forming glial cytoplasmic inclusions mainly in oligodendrocytes (2, 3) and thus designated as α-synucleinopathy (4).

The cardiovascular AF in MSA is thought to be mainly due to a progressive neurodegeneration of several areas involved in autonomic control of the cardiovascular system such as *inter alia*, cholinergic neurons in the ventrolateral ambiguous nucleus and dorsal motor nucleus of the vagus nerve (5). Many studies in MSA patients have already depicted cardiovascular autonomic impairments with a reduction in sympathetic and parasympathetic activities (6–9). It has been shown that baroreflex function was also impaired in patients with MSA (10, 11). In addition, sudden cardiac death has been reported in MSA patients, and some of them exhibited a slight increase in QTc (12). Given that QT interval depends on sympathetic and parasympathetic activities, abnormalities observed in the QTc interval may reflect the degeneration of cardioselective sympathetic and parasympathetic neurons (12).

Gender differences have been observed in MSA. Indeed, women have been reported to develop initially motor symptoms, with men exhibiting mainly cardiovascular dysfunction (13). However, some authors did not find any association between gender differences and survival (14, 15).

Mice overexpressing human α-synuclein under the control of the oligodendrocyte specific proteolipid promoter (PLP-α-syn) display aged-dependent progressive neurodegeneration and have been extensively used in the past to assess MSA-related alterations (16–19). PLP-α-syn mice have been shown to recapitulate several features of MSA such as motor dysfunction/parkinsonism (17, 18), respiratory dysfunction (20) and dysautonomia (21). The validity of transgenic mouse models of MSA is supported phenotypically with the occurrence of motor and non-motor features reminiscent of the human disease and pathologically by the accumulation of oligodendroglial inclusions containing insoluble α-synuclein, neurodegeneration in the basal ganglia (substantia nigra, striatum) as well as in other brain nuclei affected in the human disease, particularly in the brainstem (22, 23). A previous study demonstrated that 5-months old PLP-α-syn mice had reduced heart rate variability (HRV) and this could be associated with a cholinergic neurodegeneration, especially in the ambiguous nucleus and dorsal motor nucleus of the vagus nerve (24). In contrast, 9- and 12-month old mice overexpressing human α-synuclein under the control of the myelin basic protein promoter (MBP1-α-syn) showed no change in baroreflex sensitivity (BRS) and HRV under resting conditions and after pharmacological testing (25).

The underlying mechanism by which α-synuclein impacts autonomic cardiovascular dysfunction in MSA remains unclear. The main objective of the present study was to determine the effect of age and of gender in cardiovascular AF in PLP-α-syn mice.

## Materials and Methods

### Animals

Mice were maintained in a temperature and humidity controlled room on a 12:12 light–dark cycle with free access to food and drinking water. Mice expressing human WT α-syn in oligodendrocytes under the control of the proteolipid promoter (PLP-α-syn) were generated on a C57BL/6 background. WT and PLP-α-syn male and female aged 6 (5–7) and 12 (10–12) months at the beginning of the experiment were used for HRV, BRS and electrocardiography analyses.

### Heart Rate Variability

Mice were first anesthetized by mask inhalation of vaporized isoflurane (induction phase: 3%, surgical procedures: 1.5%) and were breathing spontaneously throughout the experiment. ECG signals were obtained with three limb leads and were recorded at 4 kHz sampling rate, processed and monitored (Labchart v8, AD Instruments). The R-R interval was acquired from the ECG signal over a 15-min recording. 3-min section of stable heart rate, free of noise and artifacts, were analyzed. HRV was assessed both in time, frequency and non-linear domains:

#### Time Domain

The time domain indices enable the determination of the variability in measurements of the interbeat interval (26), such as the standard deviations of the R-R intervals (SDNN) and the root-mean square differences of successive R-R intervals (RMSSD). The SDNN corresponds to all the cyclic components responsible for HRV in a defined period of recording. RMSSD expresses short-term HRV and provides information about the parasympathetic tone.

#### Frequency Domain

HRV was analyzed by the Lomb-Scargle periodogram (27, 28). The Lomb-Scargle approach is more appropriate for unevenly sampled data (29). Moreover, unlike fast Fourier transform, Lomb-Scargle does not require resampling or stationarity (27, 30, 31). Frequency domain analysis was analyzed in two separate spectral components: spectral power of low frequency (LF: 0.10–1.00 Hz) and high frequency (HF: 1.00-5.00 Hz) bandwidths. LF has been considered as an index of both cardiac sympathetic and parasympathetic tones with a dominant sympathetic component (32); while HF is thought to reflect exclusively cardiac parasympathetic tone (33). The ratio of LF to HF power (LF/HF) emphasized the sympathovagal balance (33).

### Baroreflex Sensitivity

Arterial blood pressure (ABP) was continuously monitored from the carotid artery (AD Instruments, Castle Hill, Australia). BRS was calculated from the sequence method. 5-min section of stable signals were analyzed. A baroreflex sequence was found when beat-to-beat systolic blood pressure (SBP) and R-R interval changes series in order to establish sequences of 3 or more consecutive heart beats (where either SBP increased and R-R intervals lengthened or SBP decreased and R-R intervals shortened). The BRS gain was measured by the slope of the regression line between SBP and R-R interval values obtained from each sequence. No thresholds in SBP, R-R interval or coefficient correlation (*r*) were used as previously recommended (34). The eCar software (Angers, France) was used to perform BRS analysis.

### Electrocardiography Analysis

The average ECG signal was analyzed over 3-min sections. The QT interval, corresponding to ventricular depolarization and repolarization, is identified from the beginning of the Q wave and the end of the T wave (when returned to the isoelectric line) (35). QT intervals were corrected for heart rate (QTc) using Mitchell's formula: QTc = QT/√(R-R/100) (QT and R-R interval units in ms) (36).

### Tissue Collection and Immunohistochemical Analyses

The brain was isolated, weighed, rinsed in ice-cold PBS and placed in 4% paraformaldehyde (PFA) for 48 h. Fixed brains were sectioned using the Stainless Steel Slicer Matrix with 0.5 mm coronal section slice intervals (Zivic) to collect 2 mm sections on the brainstem area. The sections were paraffin-embedded. The brainstem was processed for immunohistochemical analyses, 5 μm thick longitudinal sections from the anterior to the posterior were obtained. Slices were deparaffinized with xylene, rehydrated in decreasing concentrations of ethanol, placed in ice-cold PBS. After washing, slides were incubated with a protein-blocking agent for 60 min. Primary antibodies against TH (1:500, Abcam) and CHAT (1:500, Abcam) were incubated overnight at 4°C and after three washes with PBS+ 0.5% Tween-20, anti-rabbit HRP conjugated secondary antibody (1:10,000) diluted in blocking buffer was applied on sections and incubated for 30 min at room temperature. The sections were washed three times with PBS and 0.5% Tween-20 before being cover slipped.

### Statistics

Data were expressed as mean ± SD. Using a power calculation, we estimated that 100 animals would be sufficient to provide an 80% power and with a α risk of 5% level of significance in a bilateral hypothesis to show a difference of 20% between groups. Three-way measures analysis of variance was carried out (genotype, gender and age) using Sidak's multiple comparisons test. The comparisons were performed between groups that differ by only one factor. All statistical analyses were performed with GraphPrism 9. Differences were considered as statistically significant when *P* < 0.05.

## Results

### Heart Rate Variability

#### Time Domain

**Figure 1:**
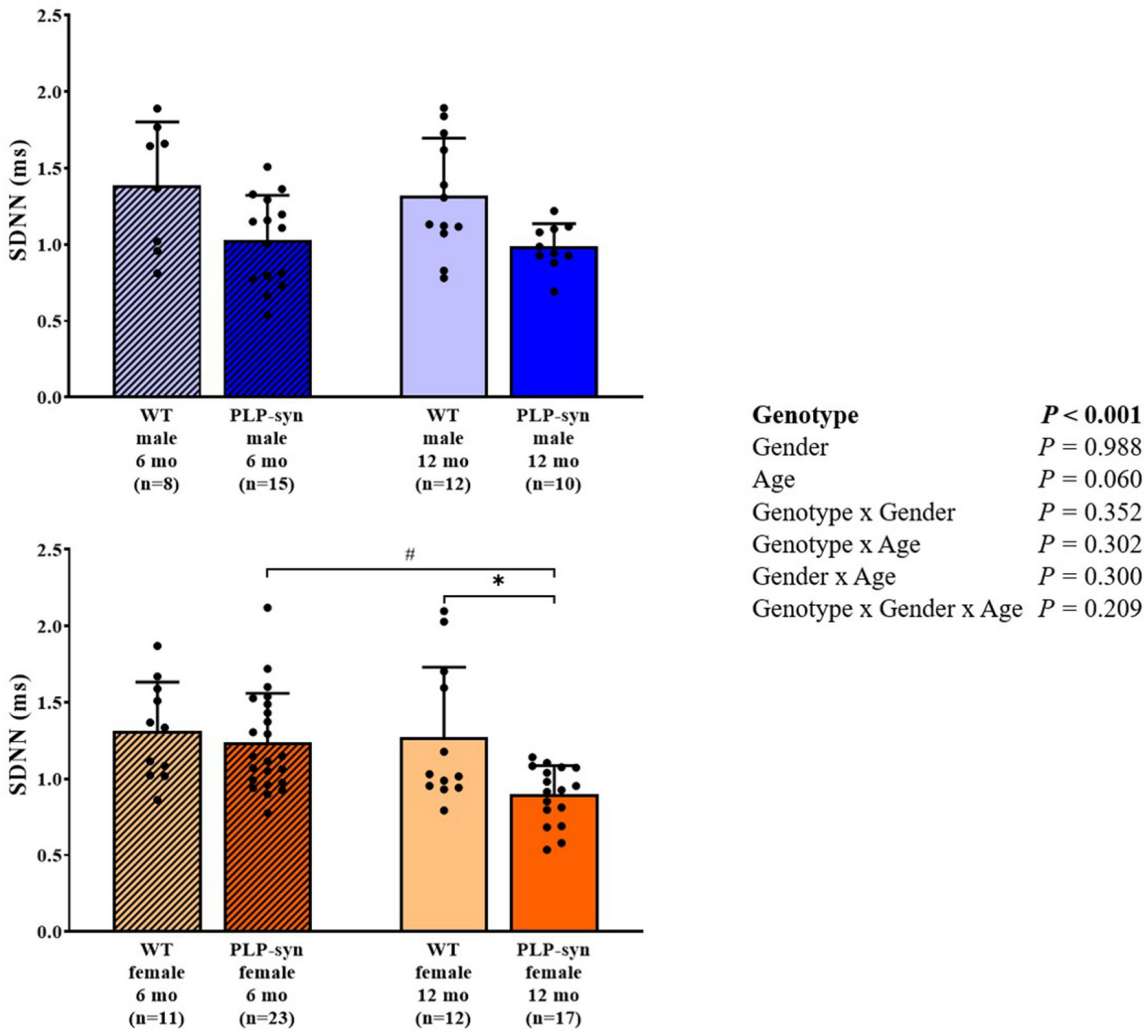
SDNN in 6 and 12 months, male and female, WT and PLP-α-syn mice. SDNN, standard deviations of normal-to-normal intervals. ^#^*P* < 0.05 vs. PLP-α-syn female 6 mo, *P < 0.05 vs. WT female 12 mo. Black circles represent individual points.

**Figure 2:**
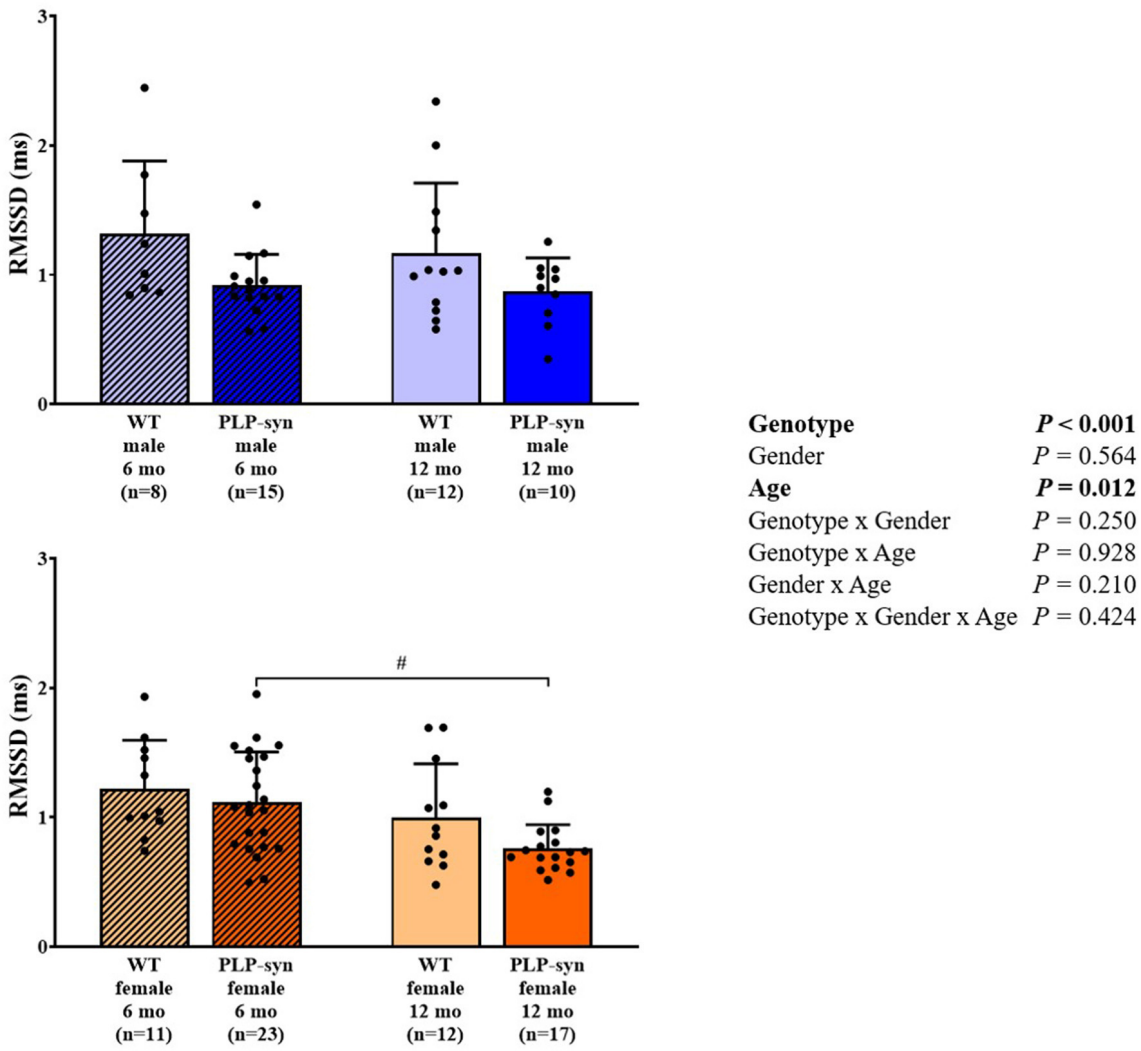
RMSSD in 6 and 12 months, male and female, WT and PLP-α-syn mice. RMSSD, root-mean square differences of successive R-R intervals. ^#^*P* < 0.05 vs. PLP-α-syn female 6 mo. Black circles represent individual points.

Three-way ANOVA indicated that there was a main effect of genotype on SDNN. *Post-hoc* comparisons showed a reduced SDNN in PLP-α-syn female mice at 12 months compared to those at 6 months (*P* = 0.015) and WT female mice at 12 months (*P* = 0.031) (Figure 1). Three-way ANOVA also showed a main effect of genotype and age on RMSSD. *Post-hoc* comparisons demonstrated that RMSSD was reduced in PLP-α-syn female mice at 12 months compared to those at 6 months (*P* = 0.043) (Figure 2).

#### Frequency Domain

**Figure 3:**
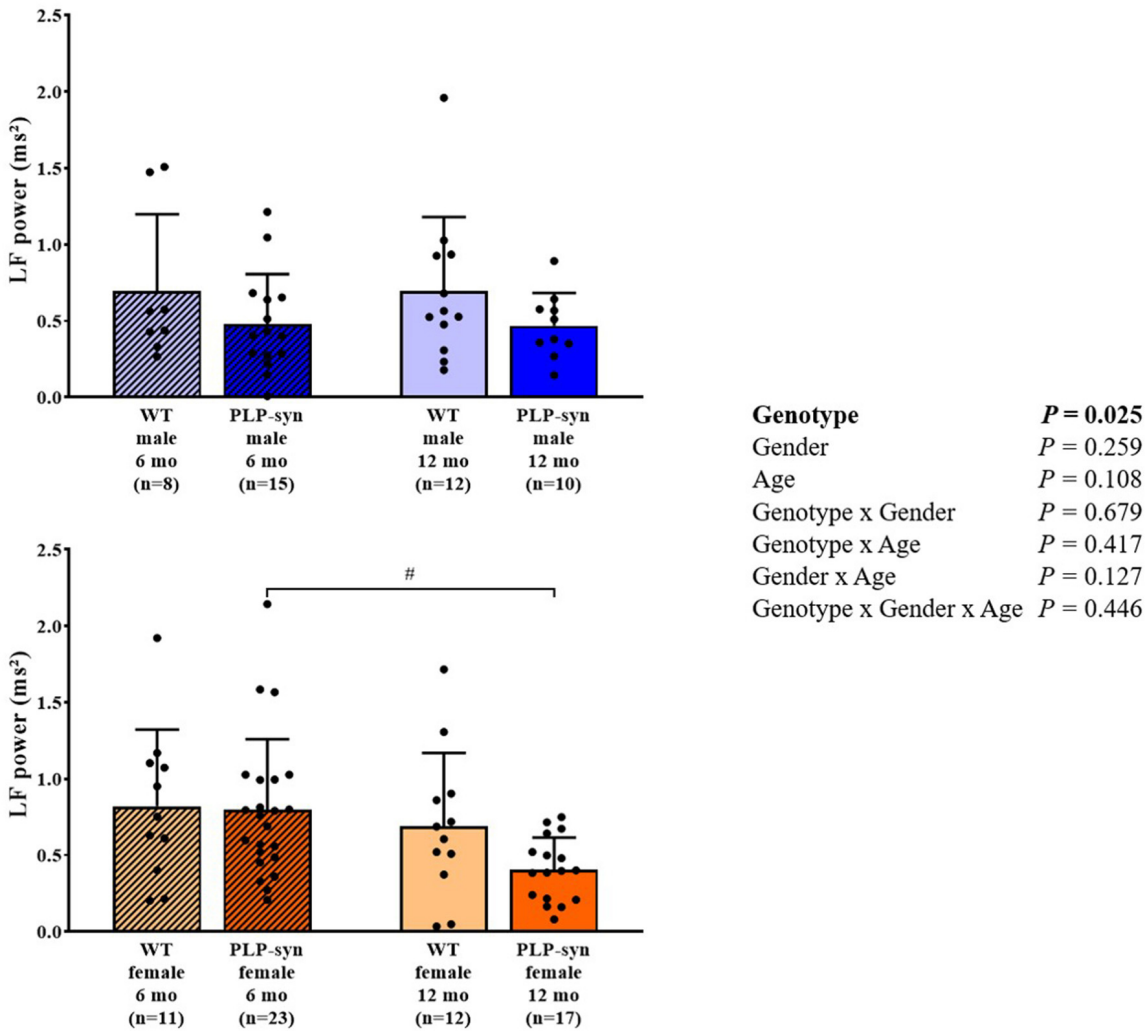
LF power in 6 and 12 months, male and female, WT and PLP-α-syn mice. LF, low frequency. ^#^*P* < 0.05 vs. PLP-α-syn female 6 mo. Black circles represent individual points.

**Figure 4:**
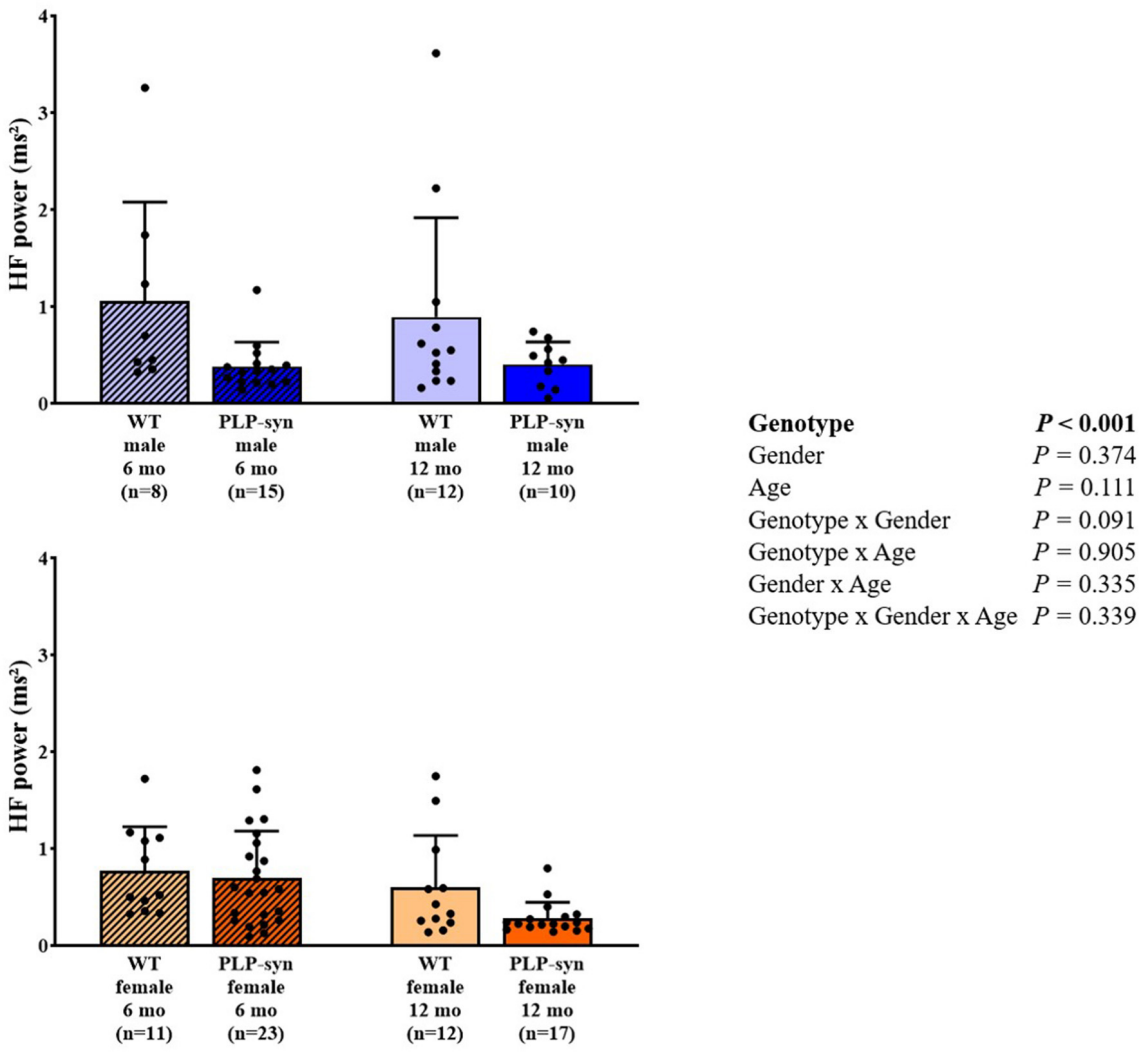
HF power in 6 and 12 months, male and female, WT and PLP-α-syn mice. HF, high frequency. Black circles represent individual points.

**Figure 5:**
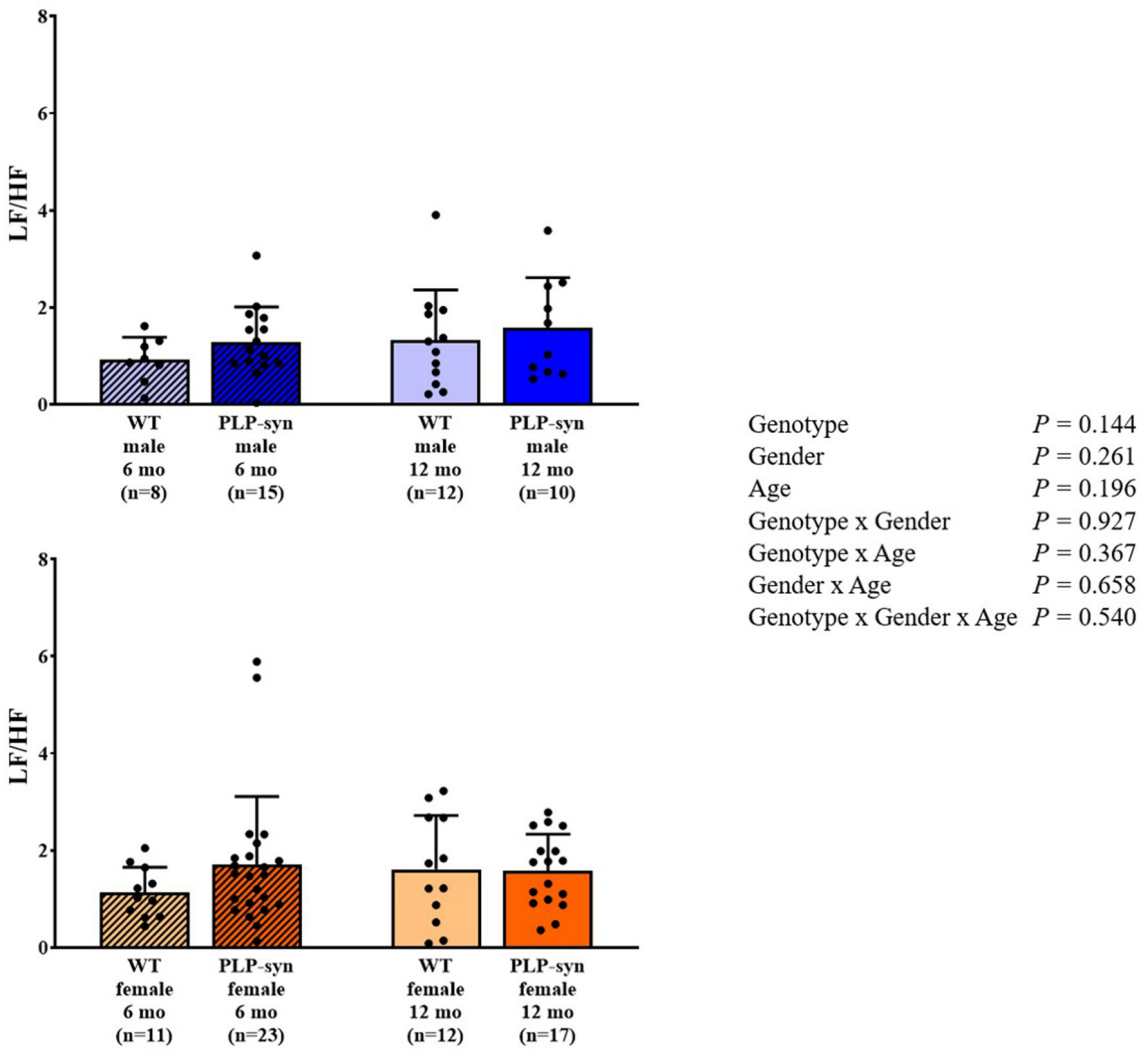
LF/HF power in 6 and 12 months, male and female, WT and PLP-α-syn mice.

As shown by three-way ANOVA, there was a main effect of genotype on LF power. *Post-hoc* comparisons depicted a reduced LF power in PLP-α-syn female mice at 12 months compared to those at 6 months (*P* = 0.041) (Figure 3). Three-way ANOVA also depicted a main effect of genotype on HF power. *Post-hoc* comparisons failed to reveal differences between groups, while we observed a trend toward a diminution in HF in PLP-α-syn male mice at 6 months compared to WT male mice at the same age (*P* = 0.075) (Figure 4). LF/HF ratio was preserved with genotype, gender and age (Figure 5).

### Baroreflex Sensitivity

**Figure 6:**
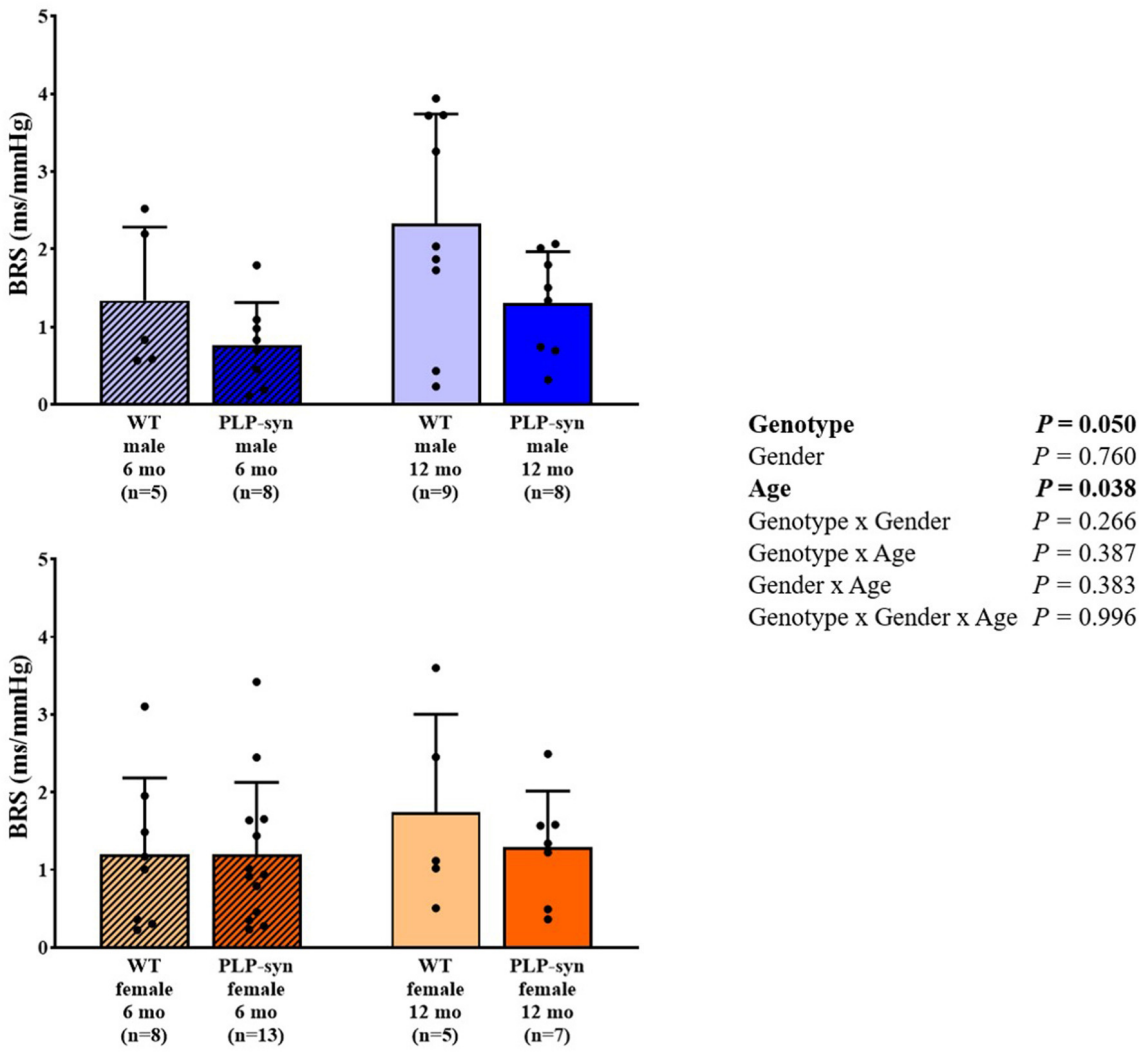
BRS in 6 and 12 months, male and female, WT and PLP-α-syn mice. BRS, baroreflex sensitivity. Black circles represent individual points.

There was a main effect of genotype and age on BRS. However, *post-hoc* comparisons did not show any significant modifications in BRS between groups (Figure 6).

### Electrocardiography Analysis

Three-way ANOVA revealed that QT and QTc intervals did not differ with genotype, gender and age (Table 1).

**Table 1:** QT and QTc data.

	**WT**				**PLP-α-syn**			
	**Male**		**Female**		**Male**		**Female**	
	**6 mo (*n* = 8)**	**12 mo** **(*n* = 15)**	**6 mo (*n* = 12)**	**12 mo** **(*n* = 10)**	**6 mo (*n* = 11)**	**12 mo** **(*n* = 23)**	**6 mo (*n* = 12)**	**12 mo** **(*n* = 17)**
**QT (ms)**	46.9 ± 5.3	44.5 ± 6.7	45.4 ± 5.8	46.1 ± 6.3	45.4 ± 3.0	44.4 ± 8.4	46.8 ± 7.7	45.4 ± 4.6
**QTc (ms)**	43.4 ± 2.7	42.2 ± 5.8	39.5 ± 5.0	40.8 ± 4.8	41.1 ± 1.8	39.7 ± 6.8	40.8 ± 5.5	41.6 ± 4.4

### Immunohistochemical Analyses

**Figure 7:**
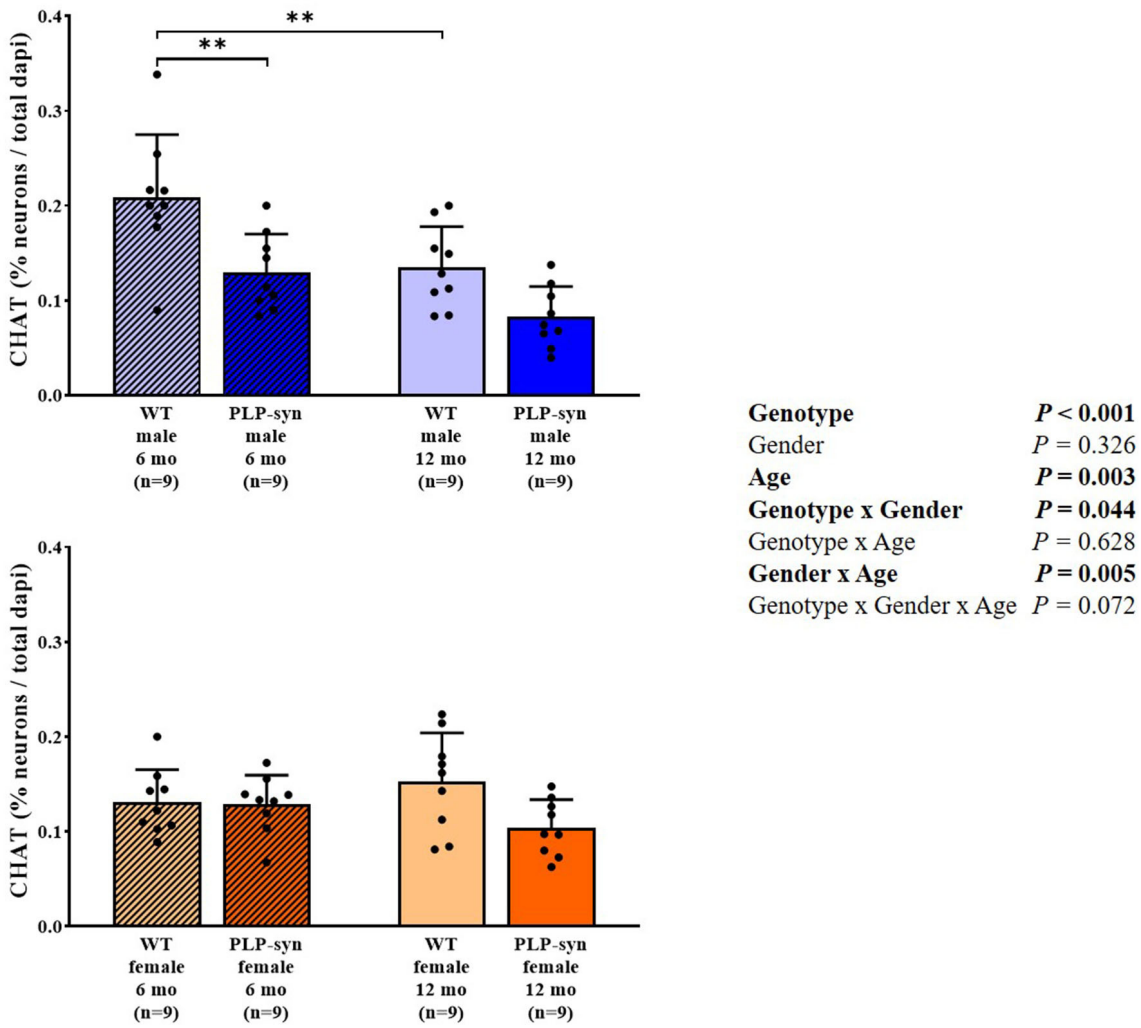
Choline acetyltransferase immunohistochemistry in 6 and 12 months, male and female, WT and PLP-α-syn mice. CHAT, choline acetyltransferase. Counts were in 100 μm^2^ areas where CHAT + neurons were counted and compared to total dapi counts. Black circles represent individual points. **P < 0.01 vs. WT male 6 mo.

Three-way ANOVA showed a main effect of genotype and age, genotype x gender and gender x age interactions on CHAT-positive cells. *Post-hoc* comparisons showed a reduction in CHAT-positive neurons in PLP-α-syn male mice at 6 months (*P* = 0.002) and WT male at 12 months (*P* = 0.006) compared to WT male at 6 months (Figure 7).

**Figure 8:**
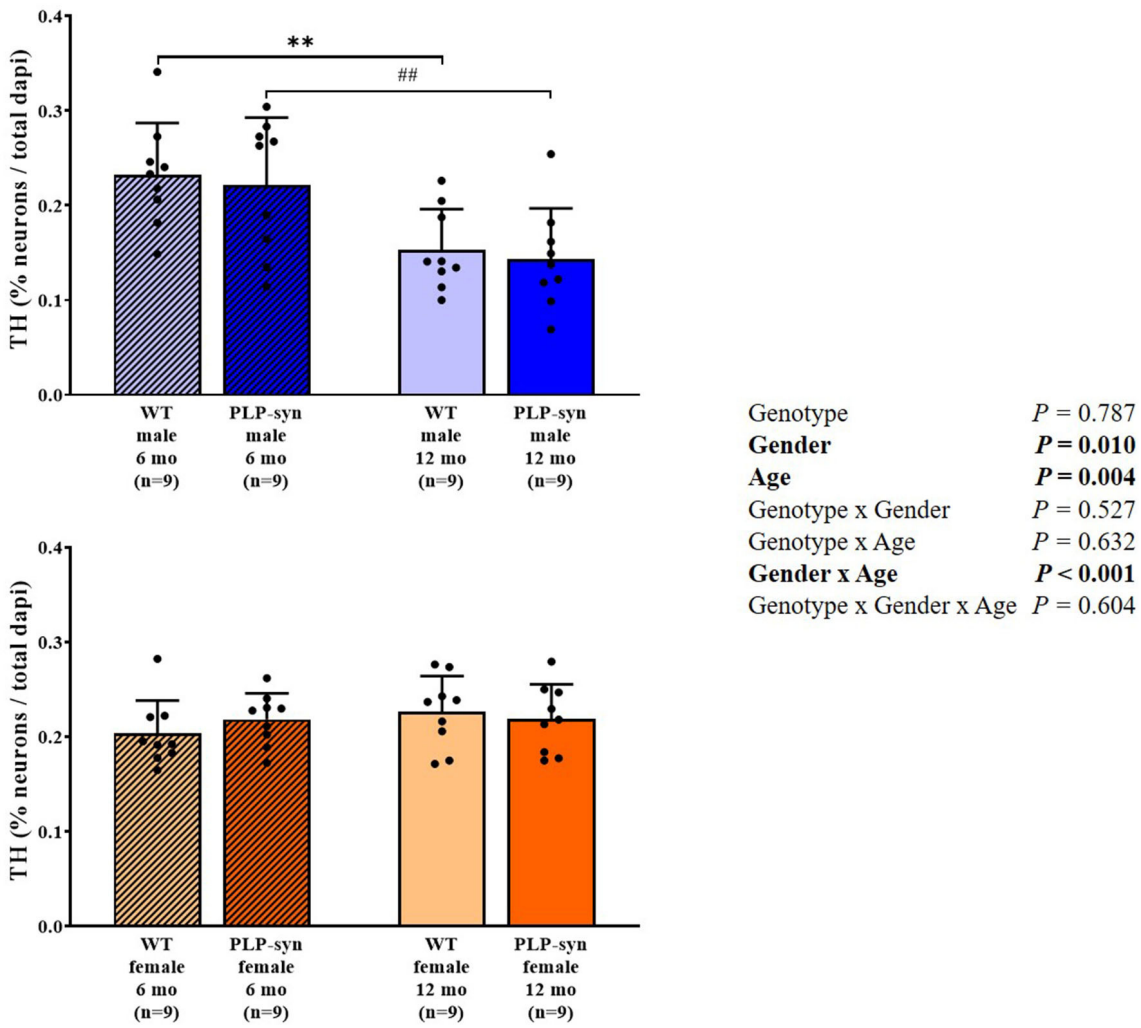
Tyrosine hydroxylase immunohistochemistry in 6 and 12 months, male and female, WT and PLP-α-syn mice. TH, tyrosine hydroxylase. Counts were in 100 μm^2^ areas where TH + neurons were counted and compared to total dapi counts. Black circles represent individual points. **P < 0.01 vs. WT male 6 mo. ^##^*P* < 0.01 vs. PLP-α-syn male 6 mo.

Three-way ANOVA also showed a main effect of gender and age and gender x age interactions on TH-positive cells. *Post-hoc* comparisons showed a reduction in TH-positive neurons in WT male at 12 months compared to those at 6 months (*P* = 0.008) and in PLP-α-syn male mice at 12 months compared to those at 6 months (*P* = 0.009) (Figure 8).

## Discussion

Our findings suggest (1) an impaired HRV in PLP-α-syn mice indicative of cardiovascular AF, whereas (2) QT and QTc intervals, which lengthening considered as an index of sudden cardiac death, were preserved in PLP-α-syn mice.

### Severe Cardiovascular Autonomic Failure

Reduced sympathetic and parasympathetic activities were observed in PLP-α-syn female mice indicating cardiovascular AF. However, cardiovascular AF was not confirmed in PLP-α-syn male mice and this was mainly due to high interindividual variability in these groups. However, the onset of cardiovascular AF seems to appear later in PLP-α-syn female than in male mice. Kuzdas et al. (24) have previously described an altered HRV in 5 months old PLP-α-syn male mice with a reduced HRV in time (RMSSD) and frequency (LF and HF) domains suggesting that sympathetic and parasympathetic activities were impaired, as is found in patients with MSA. Indeed, Diedrich et al. (37) found a dramatic fall in LF and HF components in 9 MSA patients (4 men and 5 women) compared to control patients, suggesting severe sympathetic and parasympathetic dysfunctions. Furushima et al. (8) also observed an alteration both in sympathetic and parasympathetic activities in 17 MSA patients. Furthermore, they mentioned a strong relationship between time duration and the severity of the disease. Kitae et al. (6) have also stated a diminished parasympathetic activity in 7 MSA patients (5 men and 2 women). Moreover, the progression of the disease exacerbated the decline of parasympathetic modulation. The underlying mechanism explaining cardiovascular AF in MSA is not thoroughly understood. However, one assumption is that the cardiac autonomic dysfunction observed could be due to severe neurodegeneration in several cardiovascular centers such as preganglionic sympathetic neurons in the intermediolateral cell column, and cholinergic neurons in the ambiguous nucleus and dorsal motor nucleus of the vagus nerve (5, 24, 38, 39).

### Impaired Baroreflex Function

In our study, BRS was significantly reduced in PLP-α-syn mice. A previous study performed in MBP1-α-syn transgenic mice aged 9 to 12 months fitted with radiotelemetry, showed a preserved functional autonomic phenotype (25). As suggested by these authors, overexpression of α-synuclein in this model of MSA may not be sufficient to induce cardiovascular AF. Moreover, although they did not find any changes in BRS, they found a trend of reduced CHAT-positive cells in the ambiguous nucleus which includes vagal preganglionic neurons projecting directly to the heart. In PLP-α-syn mice, Stemberger et al. (21) also demonstrated a loss of cholinergic neurons in the nucleus ambiguous. In addition, Kuzdas et al. (24) previously suggested that cardiovascular autonomic regulation may be altered by α-synuclein accumulation. It has also been described that BRS fell dramatically in 35 patients with MSA (10). Roy et al. (11) have further shown that cardiovagal BRS was impaired in 22 patients with MSA. They hypothesized that a compromised vagal reactivity and/or sympathetic denervation might be responsible for this impairment.

We also observed a slight increase in BRS with age. However, little is known about the development of baroreflex with age in mice. Ishii et al. (40) showed a cardiovascular maturation with an abrupt elevation in BRS in young adult mice compared to newborn mice.

### Preserved Ventricular Depolarization and Repolarization

In our study, we found unchanged QT and QTc intervals in PLP-α-syn mice, indicating that ventricular depolarization and repolarization were not affected. Data on QT and QTc intervals in patients with MSA are contentious. Indeed, Frongillo et al. (41) found that only 3 out of 18 MSA patients (~17%) had a prolonged QTc interval suggesting that patients with MSA did not exhibit any significant change in ventricular repolarization and dispersion. On the other hand, Deguchi et al. (12) depicted in 22 MSA patients a prolonged QTc interval compared with healthy controls. However, in the same study, the authors have also mentioned that despite a pronounced cardiovascular AF in patients with MSA, QTc interval was only slightly prolonged (+2.5%). Diedrich et al. (37) also observed in 9 patients with MSA a slight increase in QTc interval (+3.7%) compared to control subjects. In contrast in a retrospective study, Choy et al. (42) demonstrated in 36 patients with MSA that the maximum QTc interval was significantly increased compared with controls (+9.3%). The QTc prolongation in MSA patients may provide valuable information on the degeneration of cardioselective sympathetic and parasympathetic neurons, which may explain in part, sudden cardiac death in MSA patients (12).

### Cholinergic Neurodegeneration

In this study, the neuropathological examinations revealed cholinergic neurodegeneration in areas related to cardiovascular AF. We observe similar tendencies both in male and female at 12 months old of age. Consistently, a previous study showed a loss in cholinergic neurons in the nucleus ambiguous in 5-month old PLP-α-syn male mice resulting in an impaired HRV (24). In MSA patients, several studies have also described a depletion of vagal autonomic nuclei known to be involved in cardiovascular regulation (5, 39).

We observe gender- and age-related dopaminergic neurodegeneration but was not genotype-dependent. However, some studies have previously showed a loss of TH+ neurons in the substantia nigra pars compacta. Indeed, Stefanova et al. (43) showed a dopaminergic neuron loss in the substantia nigra pars compacta in 10-month old PLP-α-syn. Fernagut et al. (17) also observed dopaminergic neurodegeneration in PLP-α-syn mice aged of 18 months. Furthermore, Refolo et al. (18) showed in this same model at 6 and 12 months a dopaminergic cell loss in the substantia nigra pars compacta. The main differences observed might be explained by the fact that we assessed TH+ neurons in the medulla and not in other regions potentially affected in the human disease such as the substantia nigra pars compacta.

## Conclusion

Our results indicate an impaired HRV in PLP-α-syn female mice indicative of cardiovascular AF. In our study, BRS was significantly altered in PLP-α-syn mice in an age-dependent manner, while QT and QTc intervals were preserved. Further studies are needed to determine the pathophysiological mechanisms which lead to gender related differences in cardiovascular AF in MSA.

## Data Availability Statement

The original contributions presented in the study are included in the article/supplementary material, further inquiries can be directed to the corresponding author.

## Ethics Statement

The animal study was reviewed and approved by CEEA – 122 (Comité D'Ethique de l'US 006/CREFRE).

## Author Contributions

P-OF, WM, and AP-LT supervised the research. MK, P-OF, WM, DNA, J-MS, and AP-LT designed and performed the research, analyzed the data, wrote, and revised the manuscript. DN'G performed immunohistochemical analyses. All authors contributed to the article and approved the submitted version.

## Funding

This study was supported by Association pour le Développement de la Recherche et de l'Enseignement en Neurologie (ADREN).

## Conflict of Interest

The authors declare that the research was conducted in the absence of any commercial or financial relationships that could be construed as a potential conflict of interest.

## Publisher's Note

All claims expressed in this article are solely those of the authors and do not necessarily represent those of their affiliated organizations, or those of the publisher, the editors and the reviewers. Any product that may be evaluated in this article, or claim that may be made by its manufacturer, is not guaranteed or endorsed by the publisher.
